# Mucosal Bacteria Modulate Candida albicans Virulence in Oropharyngeal Candidiasis

**DOI:** 10.1128/mBio.01937-21

**Published:** 2021-08-17

**Authors:** M. Bertolini, R. Vazquez Munoz, L. Archambault, S. Shah, J. G. S. Souza, R. C. Costa, A. Thompson, Y. Zhou, T. Sobue, A. Dongari-Bagtzoglou

**Affiliations:** a Department of Oral Health and Diagnostic Sciences, UConn Health, Farmington, Connecticut, USA; b Department of Computer Science and Engineering, University of Connecticut grid.63054.34grid.208078.5grid.63054.34 , Storrs, Connecticut, USA; c Dental Research Division, Guarulhos University, Guarulhos, SP, Brazil; d Dental Science School (Faculdade de Ciências Odontológicas [FCO]), Montes Claros, MG, Brazil; e Department of Prosthodontics and Periodontology, Piracicaba Dental School, University of Campinas (UNICAMP), Piracicaba, SP, Brazil; f Department of Medicine, UConn Health, Connecticut, Farmington, Connecticut, USA; Geisel School of Medicine at Dartmouth

**Keywords:** *Candida albicans*, oral microbiome, pathogenesis

## Abstract

Oropharyngeal candidiasis (OPC) is the most prevalent oral infection in immunocompromised patients, primarily associated with Candida albicans. Increasing evidence points to a significant role of mucosal bacteria on the transition of C. albicans from commensal to pathogenic. In this work, we hypothesized that changes in the abundance or composition of the mucosal bacterial microbiota induced by dietary sucrose during the development of OPC can modulate C. albicans virulence. C. albicans burdens and mucosal lesions were evaluated in a mouse cortisone immunosuppression model amended with sucrose. We also analyzed the mucosal bacterial composition using 16S rRNA gene sequencing and culture methods. In immunocompetent mice, sucrose significantly increased total bacterial burdens and reduced alpha diversity, by increasing the relative abundance of mitis group streptococci. In immunocompromised mice, C. albicans infection was associated with a significantly reduced bacterial alpha diversity due to an increase in the relative abundance of enterococci. When exposed to dietary sucrose, these mice had reduced C. albicans burdens and reduced bacterial alpha diversity, associated with an increase in the relative abundance of *Lactobacillus*. SparCC correlation networks showed a significant negative correlation between *Lactobacillus* and *Enterococcus* in all *Candida*-infected mice. Depletion of lactobacilli with antibiotic treatment partially restored C. albicans burdens in mice receiving sucrose. In coculture *in vitro* experiments, mouse oral Lactobacillus johnsonii isolates inhibited growth of Enterococcus faecalis isolates and C. albicans. These results support the hypothesis that the sucrose-induced attenuation of C. albicans virulence was a result of changes in the mucosal bacterial microbiome characterized by a reduction in enterococci and an increase in lactobacilli.

## INTRODUCTION

Oropharyngeal candidiasis (OPC) is the most prevalent fungal infection in patients with immature or weakened immune systems, such as neonates (1), HIV^+^ patients (2, 3), patients undergoing intensive cancer chemotherapy (4), and transplant recipients who rely on pharmacological immunosuppression to avoid organ rejection (5, 6). These patients are susceptible to *Candida* bloodstream infection, a severe infection with mortality as high as 27% (7, 8).

Although the presence of Candida albicans and a host-permissive environment are required for the development of this infection, there is increasing evidence showing that oral candidiasis is a mixed fungal-bacterial disease and that specific interactions with certain bacterial species can promote the fungal transition from commensal to pathogenic (9). For example, in the oral cavity of leukemic children, the most abundant bacterial isolates are streptococci of the mitis group, which form robust biofilms with C. albicans isolates *in vitro* (10). Furthermore, in severely immunodeficient patients with loss-of-function mutations in the *stat3* gene, oropharyngeal candidiasis is characterized by a mucosal bacterial dysbiotic state, with the most abundant species being the mitis group member Streptococcus oralis (11). Earlier studies by our group have shown that C. albicans coaggregates with mitis group streptococci to form robust mucosal biofilms and that their interactions lead to pathogenic synergism in a murine infection model (9, 12, 13). While certain streptococcal species may contribute to C. albicans virulence in oropharyngeal candidiasis, there is also evidence that other oral lactic acid bacteria such as lactobacilli may curtail C. albicans growth and colonization *in vivo* (14).

Most of the studies on the pathogenic interactions between C. albicans and oral bacteria focused on individual bacterial species, and very few investigated the interactions between C. albicans and resident mucosal bacteria in health and disease. In our previous studies, we used Streptococcus oralis (13), which is not a member of the mouse oral microbiota, to demonstrate pathogenic synergy with C. albicans. In this work, we sought to create a favorable nutrient environment for indigenous mouse oral streptococci to thrive in order to study their effects on *Candida* virulence in immunosuppressed mice. In immunocompetent mice and humans, sucrose dietary supplementation increases the biomass of endogenous acidogenic and aciduric streptococci in the oral cavity (15–17). Sucrose is a key environmental factor that promotes coaggregation interactions between C. albicans and oral Streptococcus spp. and induces the expression of virulence genes in both organisms (18, 19). Recent multiomics analyses also suggested that a sucrose-rich environment may promote a symbiotic cross-feeding, whereby C. albicans sucrose utilization is enhanced by oral streptococcal glucosyltransferase activities (19). However, the impacts of sucrose and the resulting bacterial community changes on the virulence of C. albicans in immunosuppressed hosts are currently not known.

Here, we used a mouse immunosuppression model amended with a sucrose dietary component to allow oral streptococcal overgrowth in order to evaluate possible mutualistic relationships with C. albicans. Our hypothesis is that sucrose will lead to an overgrowth of endogenous aciduric mouse bacteria, mostly streptococcal species, which may increase the C. albicans mucosal biofilm virulence.

## RESULTS

### Sucrose increased tongue bacterial loads, reduced alpha diversity, and increased the abundance of endogenous streptococci in immunocompetent mice.

**FIG 1:**
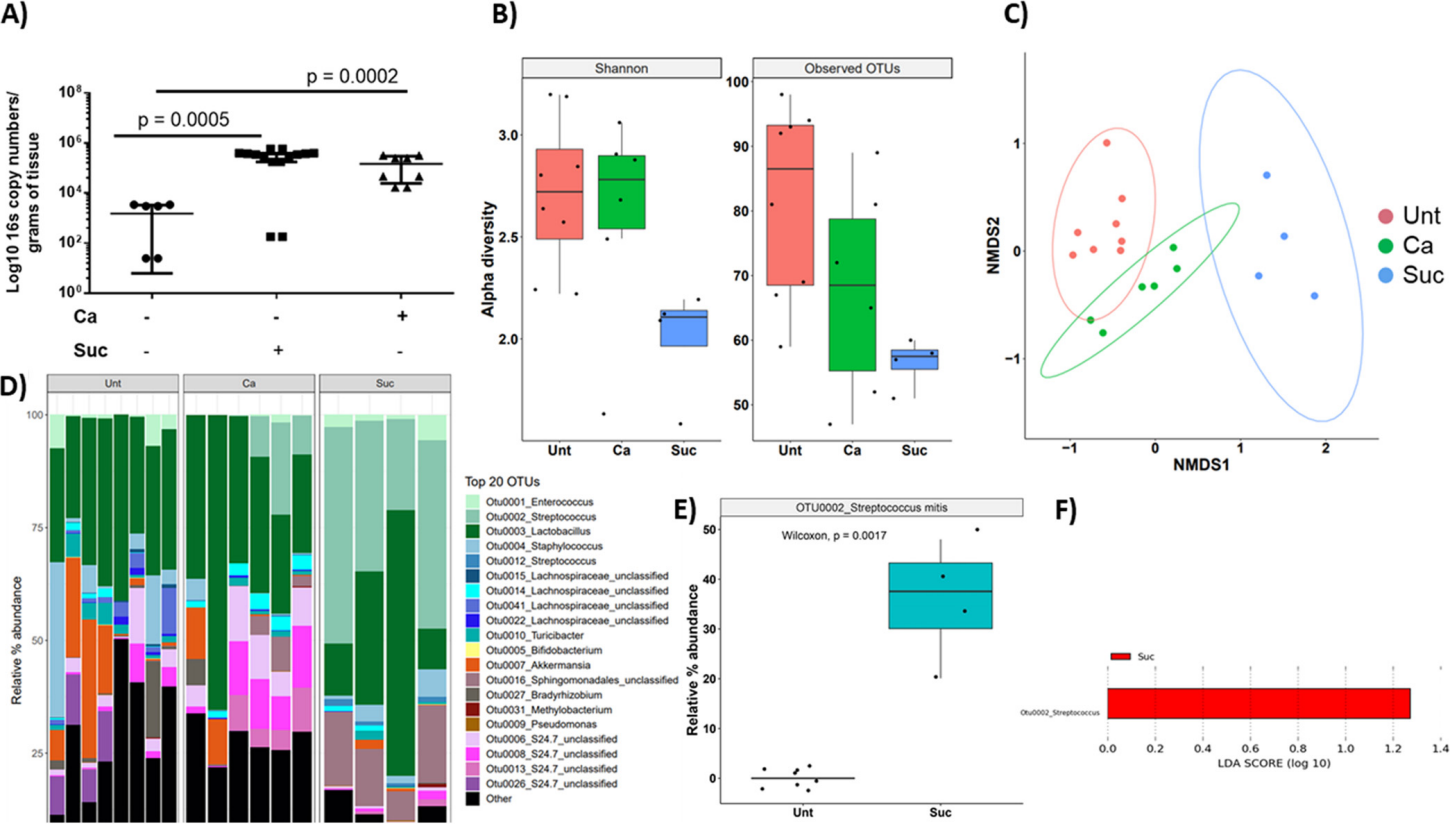
Mucosa-associated bacterial microbiota profiling in immunocompetent mice. (A) Tongue bacterial burdens compared among untreated mice (Unt), mice receiving sucrose (Suc), and mice inoculated with C. albicans SC5314 (Ca). The total bacterial biomass (log 16S rRNA gene copy numbers/g of tissue, *y* axis) was quantified by real-time qPCR after 5 days. Both sucrose (*P* = 0.005) and *Candida* (*P* = 0.0002) groups showed a significant increase in tongue bacteria compared to that in the untreated control. Data shown are from 1 to 2 independent mouse experiments, with 6 to 12 mice per group. (B) Microbial DNA was extracted from half tongues of the same mice described for panel A. The V4 hypervariable region of the 16S rRNA gene was amplified and sequenced. Box plots showing Shannon diversity index and number of observed OTUs in the three experimental groups. Mean diversity index values are shown from 4 to 8 mice in each group reaching the sequencing depth threshold as described in Materials and Methods. In mice inoculated with C. albicans (Ca), bacterial diversity did not change, but in mice receiving sucrose (Suc), bacterial diversity was significantly reduced compared to that in the untreated group (Unt) (Shannon *P* = 0.004, observed *P* = 0.008 for a comparison of sucrose versus untreated). (C) Beta diversity assessed by nonmetric multidimensional scaling (NMDS) ordination based on Bray-Curtis dissimilarities among the three experimental groups. Shown are community structures in the untreated control (Unt), sucrose (Suc), and C. albicans (Ca) groups. Results represent community structure differences at the end of the experimental period. Microbial communities clustered by type of treatment, indicating a significant effect of sucrose and C. albicans in immunocompetent mice (untreated versus sucrose, *P*_adj_ = 0.003; untreated versus *Candida*, *P*_adj_ = 0.01; *Candida* versus sucrose, *P*_adj_ = 0.008). (D) Relative abundances of bacterial genera assigned to one of the top 20 most abundant OTUs identified in mouse tongues, in each of the three treatment groups, at the end of the experimental period (*n* = 4 to 8 mice/group). Streptococcus mitis (OTU 0002) was the most abundant in mice receiving sucrose. (E) Box plot showing relative abundance of Streptococcus mitis in untreated (Unt) mice and mice receiving sucrose (Suc) in which OTU 0002 represented ∼40% of total OTUs (*P* = 0.0017). (F) Linear discriminant analysis (LDA) effect size (LEfSe) was used to identify OTUs explaining the differences between untreated (Unt) mice and mice receiving sucrose (Suc). Logarithmic LDA score threshold of 1 was chosen. Streptococcus mitis abundance was significantly different between communities.

To test whether dietary sucrose can increase the biomass of endogenous acidogenic and aciduric oral streptococci, we first analyzed its effect on immunocompetent mice. As seen in Fig. 1, dietary sucrose increased the total bacterial biomass on the tongue surface (Fig. 1A), while alpha diversity was significantly reduced (Fig. 1B). The presence of C. albicans also increased bacterial burdens on the tongue (Fig. 1A) but did not have a significant impact on alpha diversity (Fig. 1B). Interestingly, the untreated, sucrose-treated, and *Candida*-treated groups exhibited distinct bacterial global community structures as shown in nonmetric multidimensional scaling (NMDS) analyses of Bray-Curtis dissimilarities, with significant differences in beta diversity between groups (sucrose versus control, adjusted *P* value [*P*_adj_] = 0.003; *Candida* versus control, *P*_adj_ = 0.01; *Candida* versus sucrose, *P*_adj_ = 0.008) (Fig. 1C). Importantly, relative abundance estimates showed that endogenous streptococci of the mitis group, with the most abundant operational taxonomic unit (OTU) sequence (OTU 0002) matching closer to Streptococcus mitis, constituted almost 40% of the resident bacteria in the sucrose group (Fig. 1D and E), which was significantly higher than that of the untreated control group as indicated by the Wilcoxon rank test (Fig. 1E) and linear discriminant analysis (LDA) effect size (LEfSe) analysis (Fig. 1F). The increase in streptococcal burdens in response to sucrose was confirmed by quantitative culture of tongue homogenates on selective media (see Fig. S2 in the supplemental material). Specifically, sucrose led to increased CFU recoveries of endogenous streptococci on mitis-salivarius (MS) agar, whereas lactobacilli were recovered equally on De Man, Rogosa, and Sharpe (MRS) agar from samples from mice receiving or not receiving sucrose. Taken together, these data show that dietary sucrose changes the structure and composition of the resident mucosal bacterial communities, with significant enrichment of mitis streptococcal species.

10.1128/mBio.01937-21.2 FIG S2 Effect of sucrose on *Lactobacillus* and Streptococcus tongue burdens in immunocompetent mice. Sucrose was supplied in the drinking water for 5 days. Tongue homogenates were plated on selective media for validation of the bacterial microbiome sequencing data shown in Fig. 1. Selective media included MRS agar (for lactobacilli) and mitis-salivarius (MS) agar (for oral streptococci). A significant increase (*P* = 0.0027) of Streptococcus burden was noted in mice receiving sucrose compared to that in untreated mice. Download FIG S2, TIF file, 0.03 MB. Copyright © 2021 Bertolini et al. 2021 Bertolini et al. https://creativecommons.org/licenses/by/4.0/ This content is distributed under the terms of the Creative Commons Attribution 4.0 International license.

### Sucrose increased tongue bacterial loads but reduced visible biofilm lesions and fungal burdens in immunosuppressed mice with oropharyngeal candidiasis.

**FIG 2:**
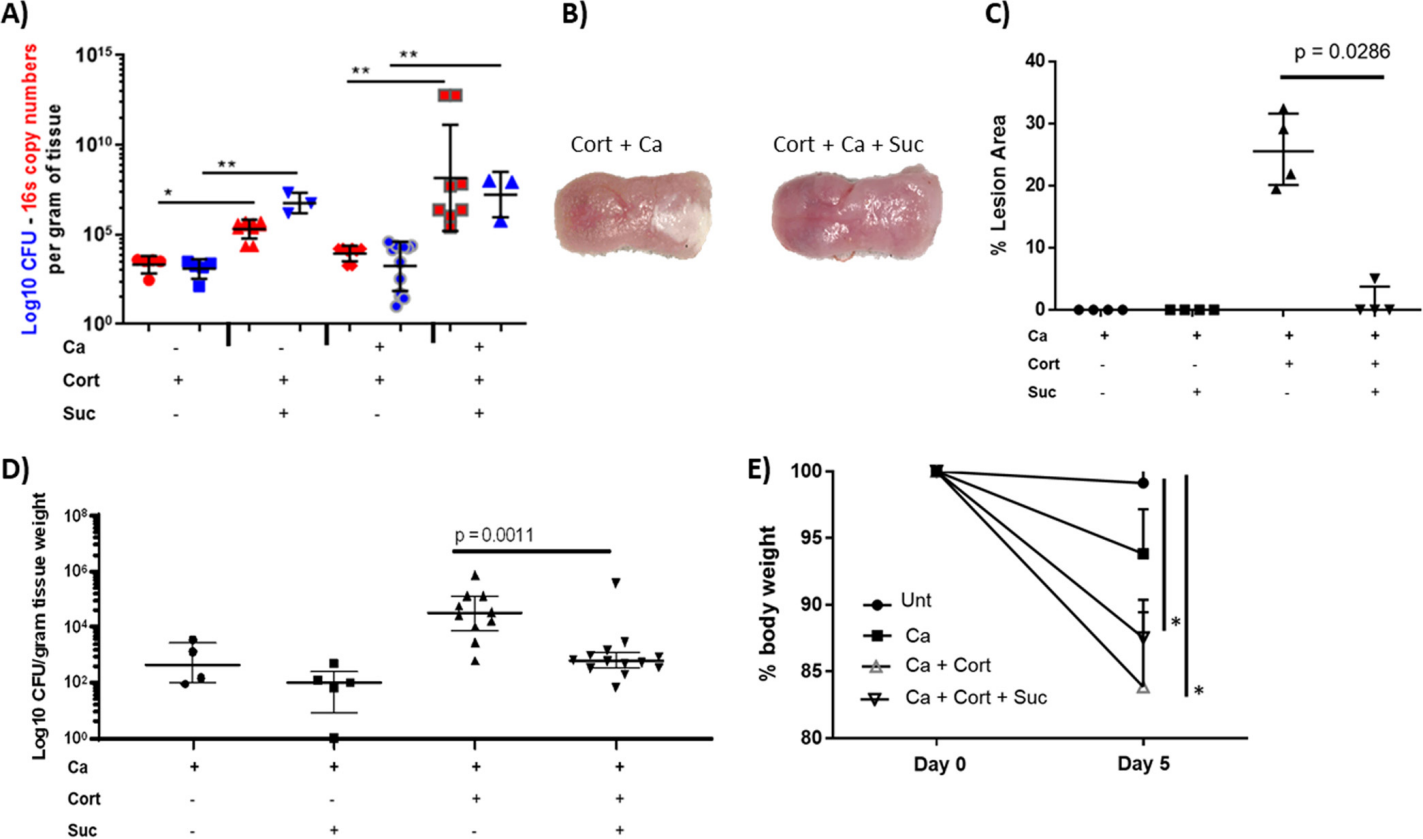
Mucosa-associated bacterial microbiota profiling in immunosuppressed mice. (A) Tongue bacterial burdens compared among control cortisone-treated mice (Cort), cortisone-treated mice receiving 5% sucrose daily in the drinking water (Suc), cortisone-treated mice inoculated with C. albicans SC5314 (Ca), and cortisone-treated mice receiving C. albicans SC5314 with sucrose. The total bacterial biomass was quantified both by real-time qPCR (red) and by total CFU counts/g of tongue homogenates (blue), plated in nonselective media on day 5. Both bacterial estimates showed a significant increase in mice receiving sucrose regardless of C. albicans infection. *, *P* < 0.05; **, *P* < 0.005 for a comparison between sucrose and no sucrose. Data shown are from 1 to 2 independent mouse experiments, with 4 to 10 mice per group. (B) Macroscopic examination of tongue mucosa of cortisone-immunosuppressed mice 5 days postinfection. The *Candida*-infected group that did not receive sucrose had a thick mucosal biofilm covering the posterior tongue surface. Minor ulcerations and absence of a robust biofilm were noted in the infected group receiving sucrose. (C) C. albicans mucosal biofilm evaluation in immunocompetent and immunosuppressed mice with or without sucrose (day 5). Tongues were digitally photographed, images were analyzed by ImageJ, and results were expressed as the percentage of dorsal area covered by visible biofilm (white area). The immunosuppressed infected mice had significantly bigger biofilms than the same group with sucrose (*P* = 0.0286). (D) Fungal burdens compared among immunocompetent and immunosuppressed *Candida*-infected groups with or without sucrose (day 5). Tongue homogenates were plated on Sabouraud dextrose agar supplemented with chloramphenicol (10 μg/ml) for C. albicans quantification. Sucrose caused a significant reduction in fungal burdens in cortisone-treated mice (*P* = 0.0011) and a modest but statistically not significant reduction in immunocompetent mice. (E) Body weight loss in immunocompetent and immunosuppressed *Candida*-infected groups with or without sucrose from baseline to day 5, expressed as percentage of initial weight (day 0), in 5 to 10 animals per group from 2 independent experiments. Error bars represent standard deviations (SDs). Weight loss in *Candida*-infected cortisone-immunosuppressed mice was somewhat reduced in the group receiving sucrose, although this was not statistically significant compared to that in mice not receiving sucrose.

We next evaluated the effect of sucrose on C. albicans virulence using the cortisone immunosuppression model. Our hypothesis was that by increasing the abundance of oral streptococci as described above, sucrose could positively influence the C. albicans mucosal biofilm virulence. In cortisone-immunosuppressed mice with or without oropharyngeal candidiasis, tongue bacterial burdens increased when sucrose was provided, as measured by CFU counts in nonselective media or by 16S gene copy numbers (Fig. 2A). However, in *Candida*-infected mice, sucrose negatively influenced the development of mucosal biofilm lesions (*P* = 0.0286) (Fig. 2B and C). In agreement with this, we noted that C. albicans mucosal burdens were significantly lower in cortisone-treated mice receiving sucrose (*P* = 0.001), and a similar trend was noted with sucrose in *Candida*-infected immunocompetent mice (Fig. 2D). At the end of the experimental period, weight loss in *Candida*-infected cortisone-immunosuppressed mice was reduced in the group receiving sucrose, although this was not statistically significant and could be due to the higher caloric intake in this group (Fig. 2E). Collectively these findings show that, contrary to our expectations, sucrose attenuated the virulence of C. albicans in the oral mucosa of cortisone-treated mice.

### Sucrose modifies the effect of C. albicans on the mucosal bacterial microbiome of immunosuppressed mice.

Given the negative effect of sucrose on C. albicans burdens, we next hypothesized that this could be due to overgrowth of antagonistic endogenous bacteria. Sucrose is efficiently metabolized by C. albicans, which converts it to glucose with an intracellular maltase (20). To rule out a direct negative effect of sucrose metabolism on C. albicans filamentous growth, in preliminary *in vitro* experiments, we determined that switching the carbon source from glucose or dextrose to sucrose did not have a significant effect on morphologic transition or hyphal length, which are important factors in mucosal virulence (21) (see Fig. S3).

10.1128/mBio.01937-21.3 FIG S3 *In vitro* effect of sucrose on C. albicans growth, morphology, and yeast-to-hyphal transition. (A) C. albicans SC5314 yeast were seeded into imaging slide wells and grown for 4 h at 37°C with 5% CO_2_ and then imaged on a Zeiss Axio Observer microscope with a 20× lens objective. Representative ×20 magnification images of C. albicans growth for 4 h on YNB (yeast-nitrogen base), YNB plus 5% glucose, or YNB plus 5% sucrose, as labeled. Images show that switching the carbon source from glucose (dextrose) to sucrose did not have a significant effect on C. albicans morphologic transition. (B) Evaluation of the percentage of each morphology (yeast, pseudohyphae, and hyphae) present at 4 h in YNB, YNB with 5% glucose (YNBgluc), YNB with 5% sucrose (YNBsuc), yeast-peptone-dextrose broth (YPD) with 5% glucose, and YPD with 5% sucrose (YPsuc), counted from microscopic images. Means ± standard deviations are plotted from 3 to 5 images per condition. Cells were marked manually in images in Fiji ImageJ and counted using the region of interest (ROI) manager. YNB media induced fewer hyphae and pseudohyphae than other tested media but without differences for glucose, sucrose, or dextrose as carbon source. (C) Hyphal lengths measured in Fiji ImageJ from images in panel A by using the ROI manager. Hyphal length was similar in glucose/dextrose versus sucrose in both media. **, *P* = 0.0083; all other comparisons, *P* > 0.05. Download FIG S3, TIF file, 0.4 MB. Copyright © 2021 Bertolini et al. 2021 Bertolini et al. https://creativecommons.org/licenses/by/4.0/ This content is distributed under the terms of the Creative Commons Attribution 4.0 International license.

**FIG 3:**
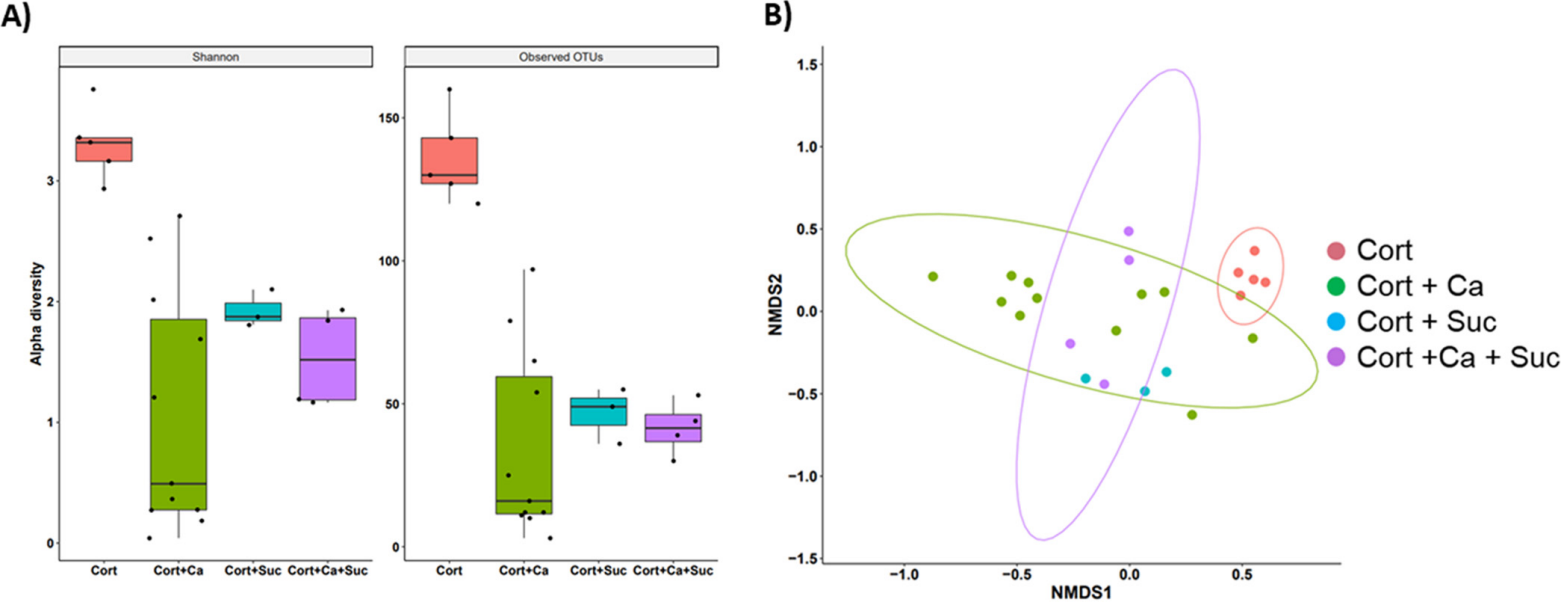
Mucosa-associated bacterial microbiota profile in immunosuppressed mice. (A) Microbial DNA was extracted from half tongues of the same mice described in the legend for Fig. 2, and the 16S rRNA gene V4 region was sequenced. Box plots showing Shannon diversity index and observed OTUs in cortisone-treated mice (Cort), cortisone-treated mice receiving sucrose (Cort+Suc), cortisone-treated mice inoculated with C. albicans SC5314 (Cort+Ca), and cortisone-treated mice receiving both C. albicans SC5314 and daily sucrose (Cort+Ca+Suc). Mean diversity index values are shown from 4 to 11 mice in each group. In all three treatments (*Candida*, sucrose, or both combined), bacterial alpha diversity was significantly reduced compared with that with cortisone treatment only. Cortisone versus cortisone plus *Candida* (Shannon *P* = 0.006, observed *P* = 0.002); cortisone versus cortisone plus sucrose (Shannon *P* = 0.036, observed *P* = 0.036); cortisone versus cortisone plus *Candida* plus sucrose (Shannon *P* = 0.016, observed *P* = 0.016). (B) Beta diversity assessed by NMDS ordination based on Bray-Curtis dissimilarities among the four experimental groups. Shown are community structures in the four groups described for panel A. Bacterial communities clustered from control cortisone-treated mice were separate from all other groups (cortisone versus cortisone plus *Candida*, *P*_adj_ = 0.003; cortisone versus cortisone plus sucrose, *P*_adj_ = 0.025, cortisone versus cortisone plus *Candida* plus sucrose, *P*_adj_ = 0.011). Sucrose-treated groups were also distinct from their respective group with no sucrose (cortisone versus cortisone plus sucrose, *P*_adj_ = 0.025; cortisone plus *Candida* versus cortisone plus *Candida* plus sucrose, *P*_adj_ = 0.008). These findings suggested that the bacterial composition was differentially altered by the presence of sucrose in immunosuppressed mice with and without oropharyngeal candidiasis.

Next, we assessed the impact of sucrose on the oral mucosal bacterial microbiome of cortisone-treated mice with or without C. albicans infection. Our data showed that both *Candida* infection and sucrose availability, separately or combined, led to a significant reduction in alpha diversity compared to that for control cortisone-treated mice (Fig. 3A). NMDS analyses also showed that beta diversity was significantly different between control cortisone-treated mice and all other groups (Fig. 3B). Beta diversity was also significantly different between the two *Candida*-infected groups with or without sucrose treatment, suggesting that the bacterial composition was altered by the presence of sucrose in mice with oropharyngeal candidiasis.

To better understand the compositional changes associated with *Candida* and/or sucrose supplementation in cortisone-treated mice, we first investigated if cortisone treatment alone, or in combination with sucrose, was associated with significantly different microbial communities. Interestingly, Streptococcus mitis (OTU 0002) was the most abundant species in cortisone-treated mice, regardless of sucrose supplementation (see Fig. S4A). Although no OTU exceeded the logarithmic LDA score threshold of 1 in LEfSe analyses, Pseudomonas was more abundant in the cortisone group and *Sphingomonadales* was more abundant in the cortisone group when sucrose was available (Fig. S4B).

10.1128/mBio.01937-21.4 FIG S4 Mucosa-associated bacterial beta diversity analysis of the 20 most abundant OTUs of cortisone-immunosuppressed mice in the presence or absence of sucrose. (A) Relative abundance of OTU sequences assigned to one of the top 20 most abundant OTUs identified on mouse tongues, in each of the two treatment groups, at the end of the experimental period (day 5, *n* = 3 to 5 mice/group). Indigenous streptococci were the most abundant genus in cortisone-treated mice regardless of sucrose supplementation. (B) Linear discriminant analysis (LDA) effect size (LEfSe) was used to identify OTUs driving the differences between cortisone versus cortisone with sucrose groups. Although no OTU exceeded the logarithmic LDA score threshold of 1, the differences seen in the principal-coordinate analysis (PCoA) plot in Fig. 3B are mostly explained by Pseudomonas in the cortisone group and by *Sphingomonadales* in the cortisone with sucrose (CortSuc) group (LDA score > 0.5 for both genera). Download FIG S4, TIF file, 0.3 MB. Copyright © 2021 Bertolini et al. 2021 Bertolini et al. https://creativecommons.org/licenses/by/4.0/ This content is distributed under the terms of the Creative Commons Attribution 4.0 International license.

**FIG 4:**
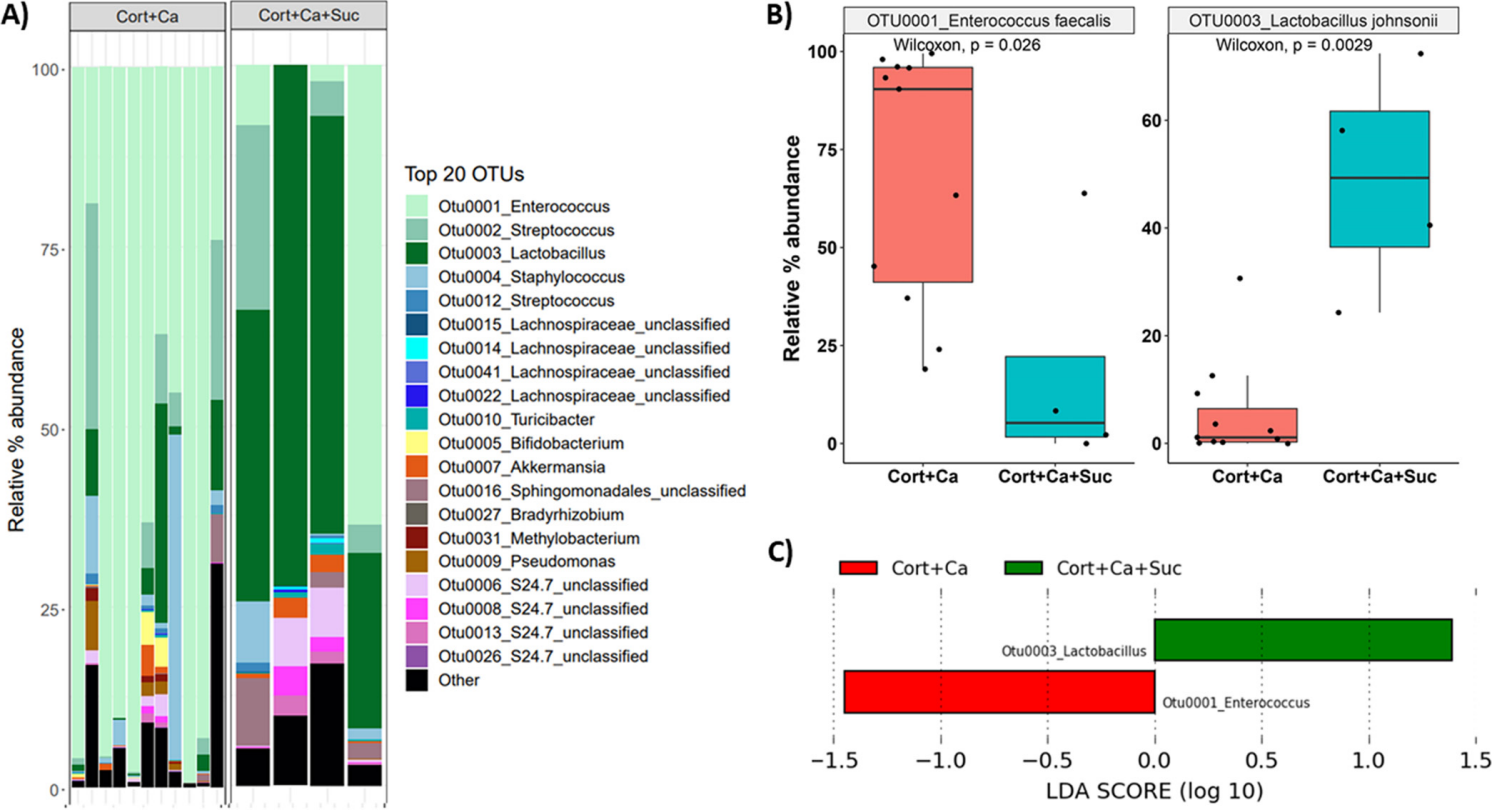
Mucosa-associated bacterial composition in cortisone-immunosuppressed mice infected with C. albicans in the presence or absence of sucrose. (A) Relative abundance of bacterial genera assigned to one of the top 20 most abundant OTUs identified on mouse tongues in each of the two experimental groups at the end of the experimental period (day 5, *n* = 4 to 11 mice/group). *Enterococcus* was the most abundant OTU in mice receiving cortisone plus *Candida* (Cort+Ca) and *Lactobacillus* was the most abundant OTU in mice receiving cortisone plus *Candida* plus sucrose (Cort+Ca+Suc) (B) Box plots showing relative abundance of the most abundant OTUs in *Candida*-infected mice with and without sucrose. *Enterococcus* represented around 90% of total OTUs in *Candida*-infected mice without sucrose (*P* = 0.026 for a comparison between infected mice with and without sucrose), and *Lactobacillus* represented around 50% of total OTUs in infected mice receiving sucrose (*P* = 0.0029 for a comparison between infected mice with and without sucrose). (C) Linear discriminant analysis (LDA) effect size (LEfSe) was used to identify OTUs explaining most of the differences between *Candida*-infected mice treated with and those without sucrose. Logarithmic LDA score threshold of 1 was chosen. *Enterococcus* and *Lactobacillus* abundance was significantly different between the bacterial communities in *Candida*-infected mice receiving or not receiving sucrose treatment.

**FIG 5:**
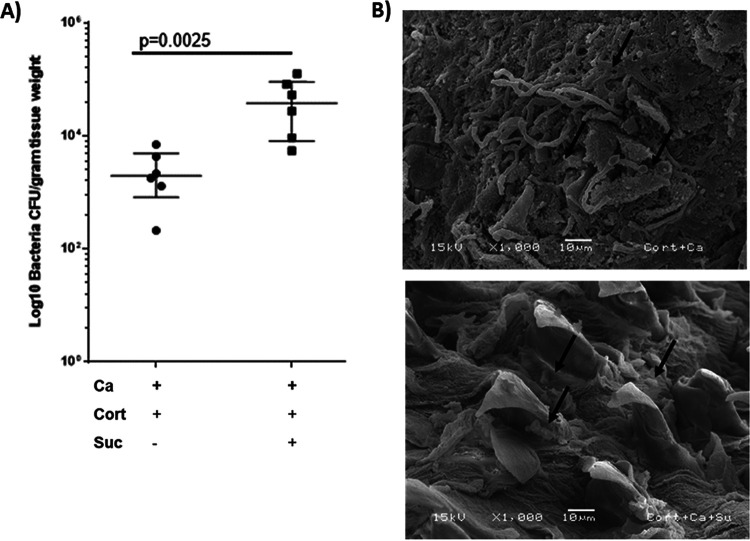
Mucosa-associated lactobacilli in cortisone-immunosuppressed mice with oropharyngeal candidiasis. (A) *Lactobacillus* burdens in cortisone-immunosuppressed mice infected with C. albicans in the presence or absence of sucrose. Tongue homogenates were plated on Rogosa agar and incubated anaerobically for 3 to 5 days for quantitative culture analysis. There was a significant increase (*P* = 0.0025) of *Lactobacillus* CFU in mice receiving sucrose. (B) Representative SEM images of the tongue surface of *Candida*-infected immunosuppressed mice with or without sucrose treatment. (Top) Biofilms formed on the tongue surface of cortisone-immunosuppressed mice infected with C. albicans. Hyphae can be seen invading the tongue surface. Arrows indicate significant amounts of cocci in direct juxtaposition to hyphae. Filiform papillae are eroded, suggesting pathology associated with infection. (Bottom) Tongue surface of *Candida*-infected mice receiving sucrose. Arrows indicate intact filiform papillae with adhering large rod-shaped bacteria, suggestive of *Lactobacillus*.

Comparing the bacterial microbiome compositions of *Candida*-infected cortisone-treated mice receiving or not receiving sucrose, we observed that sucrose significantly modulated the microbiome, with a decrease in the relative abundance of the genus *Enterococcus*, which made up to 40% to 90% of the bacterial communities in mice with oropharyngeal candidiasis in the absence of sucrose (Fig. 4A and B). In *Candida*-infected mice treated with sucrose, an expansion of endogenous lactobacilli was noted (*P* = 0.0029), which comprised 35% to 60% of the resident bacterial communities (Fig. 4A and B). This explained the finding of reduced alpha diversity coupled with the difference in beta diversity in the two infected groups in the presence versus in the absence of sucrose (Fig. 3A and B). LEfSe analyses confirmed that *Enterococcus* and *Lactobacillus* were significantly differentially enriched in these two groups (Fig. 4C). The increase in *Lactobacillus* abundance in the sucrose group was validated by quantitative culture on selective media (Fig. 5A). We conclude that in immunosuppressed mice with oropharyngeal candidiasis, sucrose caused a significant shift of the resident communities, increasing the abundance of lactobacilli and reducing the abundance of enterococci.

Representative scanning electron microscope (SEM) images of biofilms forming on the tongue surfaces (Fig. 5B) showed that in cortisone-treated mice with oropharyngeal candidiasis, C. albicans hyphae were abundant and were invading the superficial layers of the tongue mucosa, with significant erosion of the tongue papillae. Importantly, clusters of bacterial cocci were seen forming microcolonies in direct juxtaposition to fungal hyphae, suggestive of enterococci (Fig. 5B, top). However, in the *Candida*-infected group receiving sucrose, almost no C. albicans hyphae were present on the tongue surface, while filiform papillae remained intact. In this group, we also noted the presence of sparsely colonizing large rod-shaped bacteria, suggestive of lactobacilli (Fig. 5B, bottom).

**FIG 6:**
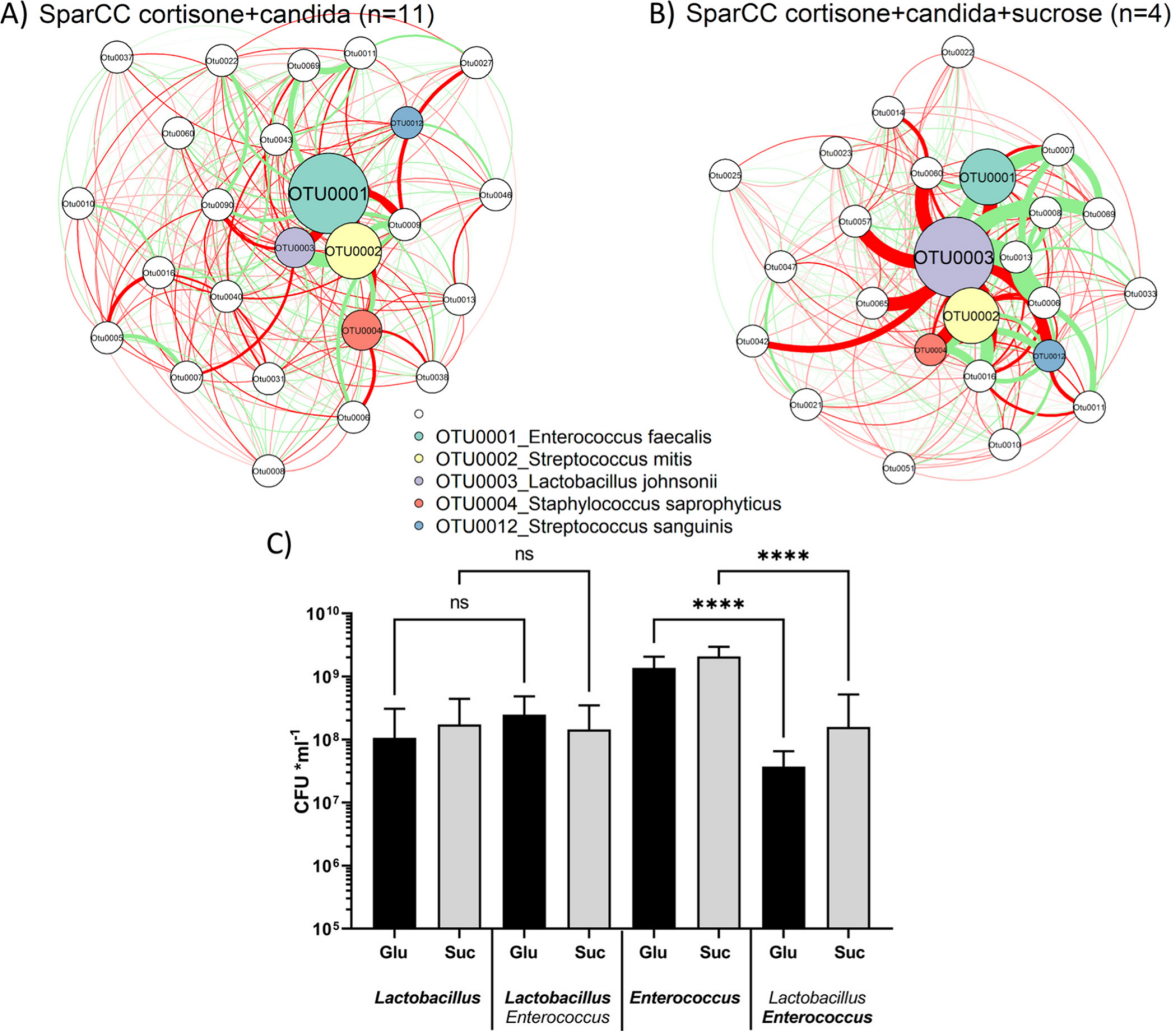
Relationship between *Lactobacillus* and *Enterococcus* as revealed by microbiome SparCC network analysis and coculture experiments. (A and B) SparCC networks highlighting the 5 most abundant OTUs (OTU0001, Enterococcus faecalis; OTU0002, Streptococcus mitis; OTU0003, Lactobacillus johnsonii; OTU0004, Staphylococcus saprophyticus; and OTU0012, Streptococcus sanguinis) in immunosuppressed *Candida*-infected mice. The five most abundant OTUs identified by species level are color coded, while “others” are white. Node size is scaled based on the overall abundance of each OTU in the microbiota. Edge width is proportional to the strength of association between each OTU pair (as measured by the correlation coefficient), red edge indicates a negative correlation, and green edge indicates a positive correlation. (A) SparCC analysis in *Candida*-infected mice; (B) SparCC analysis in *Candida*-infected mice receiving sucrose. Lactobacillus johnsonii maintained a significant negative correlation with Enterococcus faecalis regardless of sucrose treatment (*P* < 0.001, *r* = −0.64, and *P* < 0.001, *r* = −0.58, with and without sucrose, respectively). (C) Two murine isolates (L. johnsonii and E. faecalis*)* were grown in coculture in MRS broth (without dextrose) supplemented with 2% glucose or 2% sucrose anaerobically for 24 h. Aliquots were plated on Rogosa (for lactobacilli) or CATC (for enterococci) agar for quantitative culture. Bold font on the *x* axis indicates the organism whose mean CFU is shown, error bars are SDs of the means from triplicate samples in two independent experiments. CFU counts of E. faecalis were significantly lower after 24 h of coculture with L. johnsonii, regardless of the carbon source. ****, *P* = 0.001.

To understand potential interactions among the bacteria in *Candida*-infected immunosuppressed mice, in the presence or absence of sucrose, we performed SparCC network analyses, appropriate for identifying significant and potentially biologically relevant correlations between bacterial species (22). We focused on the associations between the two main genera of interest that differentiated the infected groups receiving or not receiving sucrose, i.e., enterococci and lactobacilli. We asked whether the relationships between the two corresponding OTUs (0001, Enterococcus faecalis; 0003, Lactobacillus johnsonii) were constant in the two groups, supporting biological plausibility. As expected, the presence or absence of sucrose induced distinctive cooccurrence patterns between species, with unique networks in each group (Fig. 6A and B). However, regardless of sucrose treatment, *Lactobacillus* maintained a significant negative correlation with *Enterococcus* (*P* < 0.001, *r* = −0.64 and *P* < 0.001, *r* = −0.58 with and without sucrose, respectively). This finding suggests a potential antagonistic relationship between lactobacilli and enterococci that is independent of sucrose metabolism during *Candida* infection.

To further explore this relationship, we tested the effect of lactobacilli on the growth of enterococci *in vitro*. We used two isolates from *Candida*-infected mice that were identified as L. johnsonii and E. faecalis by Sanger sequencing. Planktonic coculture experiments showed a significantly impaired growth of E. faecalis after 24 h of coculture with L. johnsonii. This growth inhibition was similar when bacteria were grown with glucose or sucrose as the main carbon source (Fig. 6C). Collectively, these data suggest that the reduction of enterococci in *Candida*-infected mice receiving sucrose may be explained by a growth inhibitory effect of lactobacilli, which were most abundant in this group.

### Reducing L. johnsonii burdens partially restores C. albicans virulence in immunosuppressed mice receiving sucrose.

**FIG 7:**
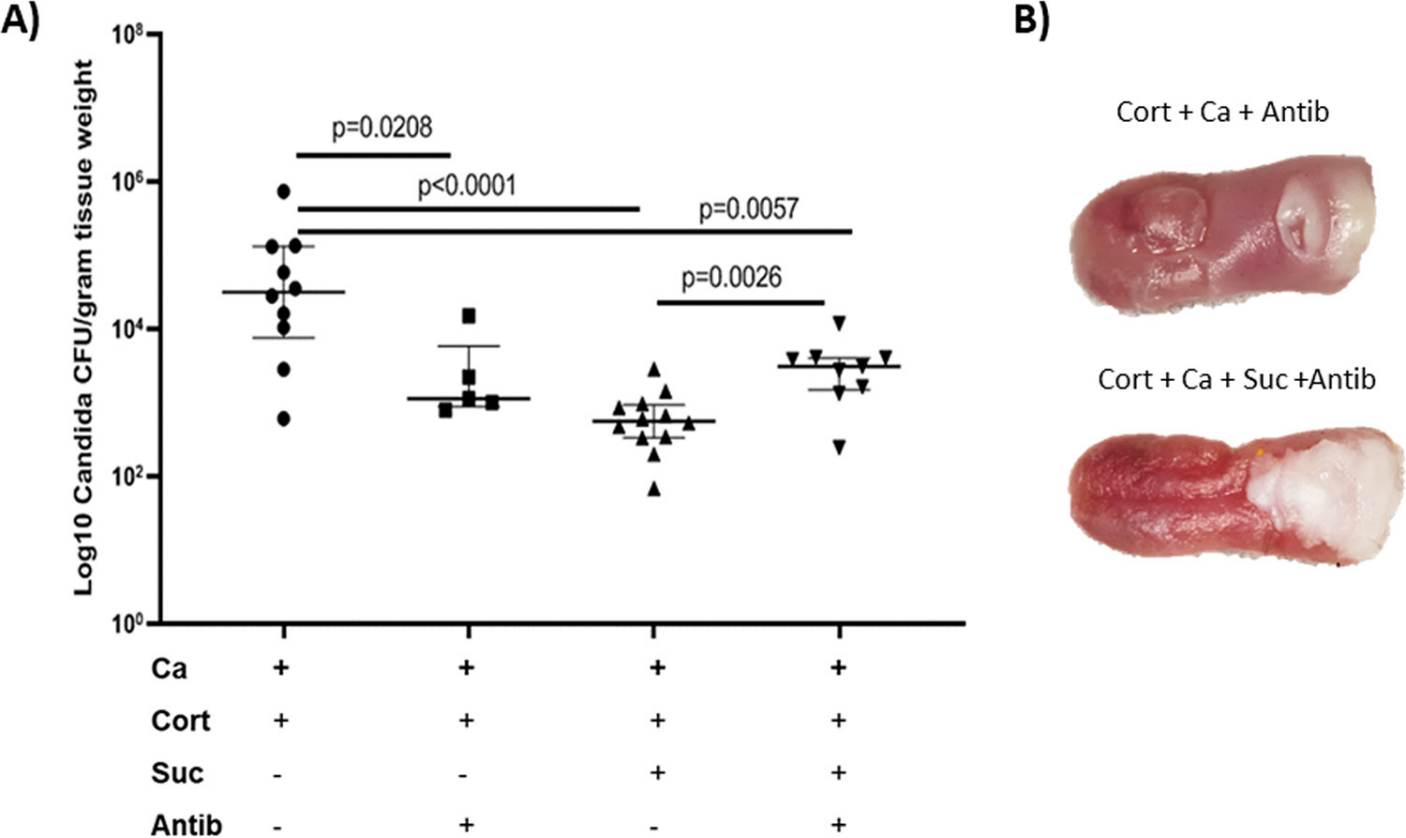
Effect of antibiotics on C. albicans virulence. (A) Fungal burdens in cortisone-immunosuppressed *Candida*-infected mice receiving sucrose or a combination of sucrose and penicillin in the drinking water. Mice were sacrificed at day 5, and tongue homogenates were plated on Sabouraud dextrose agar supplemented with chloramphenicol (10 μg/ml) for C. albicans quantification. In mice receiving sucrose, penicillin treatment resulted in significantly higher (*P* = 0.0026) C. albicans burdens than with no antibiotic treatment. In contrast, in mice not receiving sucrose, penicillin treatment significantly reduced fungal burdens (*P* = 0.0208) compared to that with no antibiotic treatment, suggesting that this treatment may reduce bacteria that have a synergistic effect with C. albicans and further underlying the differences in the bacterial compositions between sucrose-treated and untreated groups. The two antibiotic-treated groups (with and without sucrose) had similar fungal burdens. Results are from 1 to 2 mouse experiments with 5 to 12 mice/group. (B) Representative mucosal biofilm lesions on the tongues of antibiotic-treated groups with and without sucrose treatment. Both antibiotic-treated groups had similar biofilm lesions.

Because of the known negative effects of several *Lactobacillus* species on C. albicans growth (23, 24), we hypothesized that an antibiotic regimen that could reduce *Lactobacillus* species (25, 26), while sparing other lactic acid bacteria, such as streptococci and enterococci (27, 28), would at least partially restore C. albicans virulence in cortisone-treated mice receiving sucrose. In these mice, supplementing the drinking water with penicillin prevented the recovery of lactobacilli on MRS agar, whereas low-level recoveries (∼10^2^ CFU/g of tissue) on brain heart infusion (BHI) and MS agar were still possible (see Fig. S5). Recoveries on MS agar suggested that penicillin treatment allowed some streptococci to colonize these tongues. Consistent with our hypothesis, this antibiotic treatment resulted in significantly higher C. albicans burdens than the no-antibiotic treatment in mice receiving sucrose (Fig. 7A). In addition, robust mucosal biofilms were seen forming on the tongues of these mice (Fig. 7B). Interestingly, in the groups not receiving sucrose, penicillin treatment significantly reduced fungal burdens (*P* = 0.0208), possibly by reducing bacteria with synergistic virulence properties that were more abundant in the infected group not receiving sucrose (Fig. 7A).

10.1128/mBio.01937-21.5 FIG S5 Bacterial burdens in cortisone-immunosuppressed *Candida*-infected mice receiving a combination of sucrose and penicillin in the drinking water. Mice were sacrificed at day 5, and tongue homogenates were plated on BHI agar (all bacteria), MRS agar (for lactobacilli), and mitis-salivarius (MS) agar (for oral streptococci) supplemented with nystatin (250 U/ml; BHYN). Lactobacilli were not able to be recovered in this group. BHI and MS agar recoveries were still possible, showing that penicillin treatment did not clear all streptococci in this group. Download FIG S5, TIF file, 0.04 MB. Copyright © 2021 Bertolini et al. 2021 Bertolini et al. https://creativecommons.org/licenses/by/4.0/ This content is distributed under the terms of the Creative Commons Attribution 4.0 International license.

**FIG 8:**
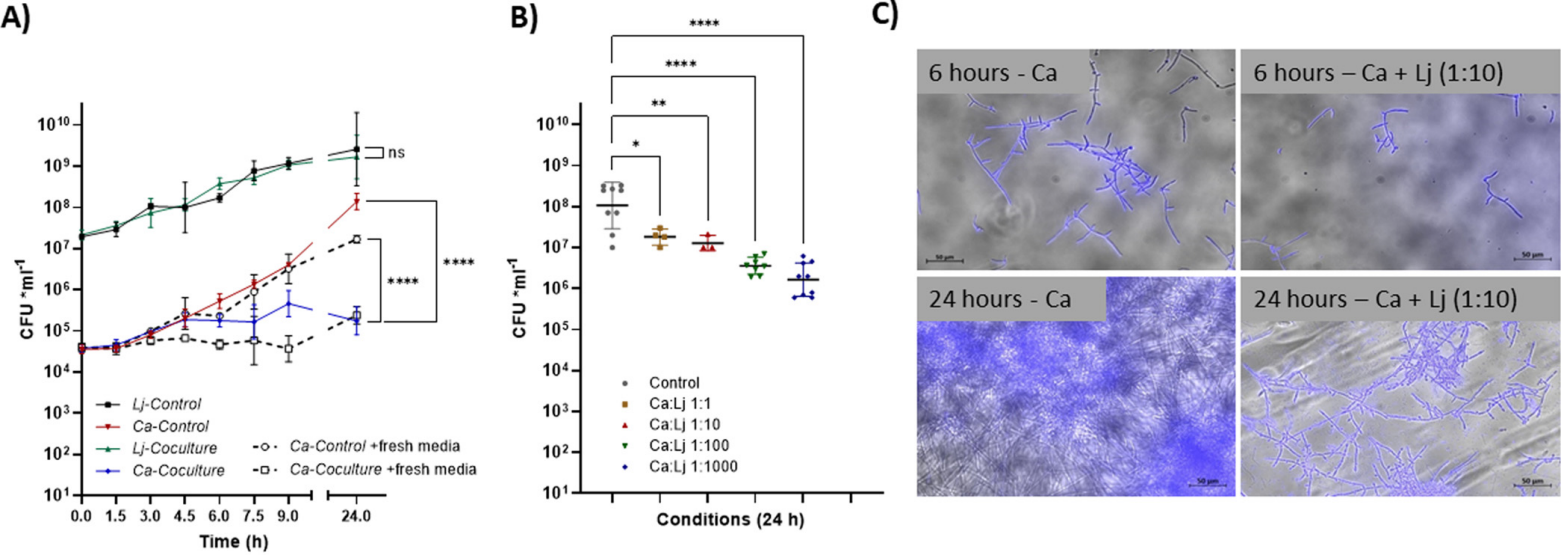
Effect of Lactobacillus johnsonii isolated from sucrose-treated mice on C. albicans planktonic and sessile growth *in vitro.* (A) A murine L. johnsonii isolate was grown alone or in combination with C. albicans in 5 ml MRS broth aerobically in 5% CO_2_ for up to 24 h. Aliquots (100 μl) were collected every 90 min, plated in triplicates, and incubated anaerobically on MRS agar (for lactobacilli) or aerobically on Sabouraud dextrose agar supplemented with 10 μg ml^−1^ of chloramphenicol (for *Candida*) for CFU determinations. In some experiments, fresh media were added after aliquot collection at each time point (dotted lines). There was impaired planktonic growth of C. albicans (but not L. johnsonii) after 6 h of coculture with L. johnsonii, regardless of medium addition. This growth inhibition became even more striking after 24 h. (B) A significant dose-dependent inhibition was noted for fungal planktonic growth after 24 h with fungal/bacterial (Ca:Lj) cell ratios ranging from 1:1 to 1:1,000. *, *P* < 0.05; **, *P* < 0.01; ****, *P* < 0.0001. (C) To assess the effect of L. johnsonii on the ability of C. albicans to grow as a biofilm, microorganisms were inoculated alone or together in 8-well slides in rich biofilm medium (RPMI, 10% BHI, 10% FBS) previously optimized for biofilm growth of *Candida* with other lactic acid bacterial species. L. johnsonii and C. albicans were seeded at 10:1 bacterial/fungal cell ratio and incubated for 6 h or 24 h. To visualize *Candida*, calcofluor white was added 5 min prior to imaging with a fluorescence microscope. Significant inhibition of hyphal growth after 6 h and 24 h of coculture with L. johnsonii was noted, while at the end of the incubation period, media were only mildly acidified (pH ∼6). Shown is one representative image from three independent experiments.

To further support the hypothesis that the reduced *Candida* virulence in mice receiving sucrose was due to antagonistic interactions between *Candida* and endogenous lactobacilli, we tested the direct effect of L. johnsonii isolated from these mice on C. albicans planktonic and sessile growth *in vitro*. Our results showed an impaired planktonic growth of C. albicans after 6 h of coculture with L. johnsonii. This growth inhibition became even more striking after 24 h and was not significantly affected by adding fresh medium at each time point, suggesting nutrient competition was not responsible for this effect (Fig. 8A). Additionally, the effect of different cell concentrations of L. johnsonii on C. albicans was assessed, and a dose response was noted for fungal growth inhibition after 24 h (Fig. 8B). In these planktonic coculture experiments, performed in MRS containing dextrose, the media were strongly acidified after 24 h (pH ∼3.5), which can inhibit *Candida* growth (29, 30). In MRS without dextrose, coculture of the two organisms caused a lower but significant inhibition in C. albicans growth (not shown), while the media were not significantly acidified after 24 h (pH ∼6.5). Lastly, assessing the effect of L. johnsonii on C. albicans sessile growth, we noted significant inhibition of hyphal growth after 6 h and 24 h of coculture with L. johnsonii (Fig. 8C). In the biofilm coculture experiments, performed in rich biofilm media, the media were only mildly acidified after 24 h (pH ∼6). Collectively, these *in vitro* experiments suggested that the negative effects of L. johnsonii on C. albicans growth may be both dependent and independent of pH. Taken together, these results support the idea that the negative effect of sucrose on C. albicans virulence was a consequence of expansion of antagonistic lactobacilli on the oral mucosa.

## DISCUSSION

Although C. albicans is the major opportunistic pathogen isolated in oropharyngeal candidiasis, the pathogenesis of this infection is highly complex due to the interactions between this fungus and the rich bacterial microbiota present in the oral cavity. Here, we showed that local environmental factors, such as increased availability of sucrose, can change the local composition of microbial communities. Under these conditions, bacterial community changes may involve overgrowth of endogenous lactobacilli, which curtail C. albicans growth or filamentation, thus attenuating virulence. In addition, most species of *Lactobacillus* are capable of preventing C. albicans adhesion to host cells in a contact-dependent manner, which maintains C. albicans as a commensal (29). Although unexpected in our model, our results also support the existing evidence regarding the use of *Lactobacillus* spp. as probiotics to prevent or treat mucosal *Candida* infections (30), as lactobacilli produce several antifungal metabolites (31). Interestingly, others have shown that L. johnsonii can interact directly with *Candida* species and degrade the fungal cell wall via chitinase-like and mannosidase-like activities (32). This may explain the absence of yeast or hyphae on the tongue surface by SEM imaging.

The availability of fermentable carbohydrates, such as sucrose, has a strong influence on the ecology of the oral microbiota, leading to decreased richness, mainly due to a significant increase in abundance of several streptococcal species (16, 33, 34). Because mitis group streptococci have been shown to have a synergistic relationship with C. albicans (35), we hypothesized that sucrose-mediated increase of these bacteria on the oral mucosa could increase *Candida* virulence. Interestingly, a high abundance of Streptococcus spp. was observed in cortisone-treated uninfected mice, even without sucrose, which may create a favorable environment for *Candida* virulence in this host immunosuppression background. Our microbiome composition data are in accordance with the recently published 16S rRNA gene draft genome database for murine oral bacterial communities, which showed two major oral bacterial lineages, including Streptococcus and *Lactobacillus* (36).

Once *Candida* was introduced in cortisone-treated mice, a shift toward *Enterococcus* was observed, mimicking what was previously reported by our group using a mouse cancer chemotherapy OPC model in which endogenous enterococci constituted almost 90% of the resident bacteria (37). As in the chemotherapy model, all of the *Enterococcus* isolates from mice with OPC were identified as E. faecalis. Such E. faecalis-dominated oral microbiome shifts have also been reported in long-term transplantation-related immunosuppressed patients (38) and recipients of allogeneic hematopoietic cell transplants preceding episodes of invasive candidiasis (39). Although E. faecalis OG1RF secretes a protein (EntV) with anti-*Candida* function (40), we previously showed that murine oral E. faecalis isolates express lower levels (compared to strain OG1RF) of the gelatinase needed for posttranslational processing and optimal EntV activity (37). This may explain the abundance of hyphae in the oral mucosa of *Candida*-infected immunosuppressed mice where enterococci were most abundant.

In the murine immunosuppression model, sucrose caused a shift not toward Streptococcus, as anticipated, but toward *Lactobacillus.* It is possible that lactobacilli, which also thrive in an acidic environment caused by fermentation of sucrose (41, 42), produced bacteriocins or metabolites resulting in Streptococcus and *Enterococcus* growth inhibition (43, 44). The negative effects of lactobacilli on the growth and biofilm formation of Streptococcus spp. have been widely documented in the literature and are associated with the production of bacteriocins or bacteriocin‐like polypeptides that have an inhibitory effect on the growth and biofilm formation of other Gram‐positive bacteria (45) and are active between pH 3 and 5 (46, 47). Along the same lines, *Lactobacillus* activity was recently reported against E. faecalis mediated by lipoteichoic acid (48) in addition to a secreted bacteriocin (49). This may explain the negative correlations between lactobacilli and enterococci revealed in SparCC analyses and the growth inhibitory effect of L. johnsonii on E. faecalis *in vitro*. However, little is known about Lactobacillus johnsonii and its interactions with streptococci or enterococci.

In a murine C57BL/6 colitis model, L. johnsonii restored the imbalance between aerobic and anaerobic populations and resulted in a significant reduction in inflammatory markers and increases in anti-inflammatory cytokines, TLR9 expression, and chitinase-like protein-1 activity, which contributed to *Candida* clearance (32). Strain genetics and commercial source can influence the dominant *Lactobacillus* species in the mouse oral microbiome (50), with L. johnsonii being the dominant oral *Lactobacillus* species in C57BL/6 mice from Jackson labs (51). Thus, our results showing this species to be most abundant may have been influenced by the choice of mouse strain and source. However, sucrose utilization as a carbon source is conserved among oral *Lactobacillus* species (52), suggesting that our results may extend to other oral species having antagonistic relationships with C. albicans.

In this work, we used penicillin to reduce the *Lactobacillus* burdens during infection in mice receiving sucrose. Our choice of antibiotic was based on evidence that *Lactobacillus* isolates from the oral cavity of rodents are susceptible to penicillin and completely resistant to other antibiotics such as streptomycin, gentamicin, and ciprofloxacin (26). In general, *Lactobacillus* species are more susceptible to beta-lactam cell wall synthesis inhibitors, such as penicillins and β-lactamase inhibitors, than to other antibiotics (53). On the other hand, it was expected that streptococci will have some resistance to penicillin (28), since the rate of resistance for penicillin in these organisms is as high as 61.2% (54). While resistance of *Enterococcus* species to beta-lactam antibiotics is well documented, there is a wide range of sensitivity, even in highly resistant strains (55, 56). Thus, while penicillin is relatively effective against lactobacilli, it may also reduce the growth of susceptible and potentially synergistic Gram-positive cocci such as streptococci and enterococci. This may explain the decrease in *Candida* burdens in mice receiving penicillin during infection in the absence of sucrose. Thus, antibiotics may positively or negatively affect *Candida* oral mucosal virulence depending on the type and duration of antibiotic treatment and also on the local composition of the bacterial microbiota.

To the best of our knowledge, this is the only study that attempted to alter the composition of the mucosal microbiota by providing an alternate bacterial nutrient source with the intent to modify fungal virulence. We report that the dominant type of carbon source combined with fungal infection can cause different mucosal bacterial shifts than either parameter alone. While most studies evaluated the probiotic effect of *Lactobacillus* species against *Candida* colonization in the gut or vaginal mucosa, we present evidence supporting a possible role of lactobacilli in ameliorating OPC. Future studies should consider the environmental and microbiome differences in the upper and lower gastrointestinal niches in this model and further address the direct and indirect mechanisms of L. johnsonii and C. albicans interactions in order to better dissect mechanistic pathways that can keep C. albicans commensal in the complex oral microbial environment.

## MATERIALS AND METHODS

### Strains and growth conditions.

C. albicans SC5314 is a bloodstream isolate (57) that forms robust oral biofilms. C. albicans was maintained in yeast extract-peptone-dextrose (YPD) agar and grown in YPD broth aerobically at 30°C on a shaker overnight prior to all experiments (58). Hyphal transformation in media (yeast nitrogen base [YNB] or YPD) containing different carbon sources (glucose or dextrose versus sucrose) was quantified microscopically after 4 h of growth, and hyphal length was assessed using ImageJ software. *Lactobacillus* and *Enterococcus* isolated from the tongues of mice infected with C. albicans were grown anaerobically on De Man, Rogosa and Sharpe (MRS) or BHI agar and cultivated to early log phase in MRS or BHI broth anaerobically at 37°C overnight prior to experiments.

### Mouse oral infection model.

Six- to seven-week-old female C57BL/6 mice were purchased from Jackson Laboratories (animal protocol 102251-0323). C. albicans infection has been described in detail elsewhere (35). Briefly, mice were immunosuppressed using cortisone acetate (225 mg/kg body weight, subcutaneously) on the first and third days of the experiment. On the second day, mice were infected with a cotton pellet saturated with 100 μl of C. albicans suspension (6 × 10^8^ cells/ml) placed subglossally for 2 h under anesthesia (ketamine-xylazine (90 to 100 and 10 mg kg^−1^ of body weight, respectively, via intramuscular injection). Fresh yeast suspension (6 × 10^6^ yeast organisms ml^−1^) was provided daily in drinking water. Mice were sacrificed on day 5, and tongues were harvested for microbial analyses and macroscopic or microscopic tissue evaluation. Body weight loss was monitored as a sign of animal morbidity and expressed as percentage of initial weight. In some experiments, mice received antibiotics (penicillin, 1.5 mg/ml) in their drinking water, starting 3 days prior to fungal inoculation and continuing throughout the experimental period. Where indicated, water was also supplemented fresh daily with 5% sucrose, starting on day one.

### Quantification of mucosa-associated C. albicans and bacteria.

Excised tongues were weighed and homogenized. Undiluted and diluted homogenates were plated aerobically at 37°C for 24 h on Sabouraud dextrose agar supplemented with chloramphenicol (10 μg/ml) for C. albicans quantification. Cultivable bacterial isolates were quantified by plating aliquots of the same homogenates on brain heart infusion (BHI) agar (for total bacteria), MRS agar (for lactobacilli), and mitis-salivarius (MS) agar (for streptococci) supplemented with nystatin (250 U/ml) (59). Bacterial cultures were incubated anaerobically for 2 to 5 days (60). Bacterial genomic DNA from mouse tongues was prepared using an overnight lysis protocol optimized for oral mucosal microbiome characterization in murine models (51). Tissues were processed the next day using the Qiagen DNA blood and tissue minikit. DNA quantity and quality were evaluated using a NanoDrop. Total bacterial biomass (16S rRNA gene copy numbers) was quantified by quantitative PCR (qPCR) with primers, TaqMan probe sequence, and thermal cycling procedures as previously published (61).

### Visualization of mucosa-associated biofilms.

After excision, tongues were digitally photographed, and images were analyzed by ImageJ to calculate the percentage of dorsal surface covered by visible biofilm (white area). To evaluate hyphae and yeast morphology on the dorsal tongue surface, a scanning electron microscope (SEM) was used, as described in detail previously (17).

### Bacterial DNA sequencing.

DNA extracted from tongues was quantified using the Quant-iT PicoGreen kit (Invitrogen). 16S rRNA genes were amplified in triplicates using 30 ng of extracted DNA as the template. The V4 region was amplified using 515F and 806R primers with Illumina adapters and bar codes on the 3′ ends (62). PCR products were pooled for quantification and visualization using the QIAxcel DNA fast analysis kit (Qiagen). Pooled PCR products were then processed using the Mag-Bind RxnPure Plus kit (Omega Bio-tek) according to the manufacturer’s protocol to include only sequences between 250 and 400 bp. The cleaned pool was sequenced on the MiSeq using a v2 2 by 250-base-pair kit (Illumina). Sequences were processed according to a standard pipeline (63) and classified using Mothur’s version of the Ribosomal Database Project classifier (Mothur 1.39.5) (64).

### Bacterial microbiome sequence analyses.

For operational taxonomic unit (OTU) analyses, sequences were clustered using a 97% similarity cutoff and classified up to the genus level based on the consensus taxonomy. Alpha and beta diversity statistics were calculated by subsampling 1,000 reads per sample. Alpha diversity was measured via Shannon diversity index and OTU richness in each sample. The relative abundance of OTUs of main genera was plotted by using stack bar graphs, including the 20 most abundant OTUs in each group. Nonmetric multidimensional scaling (NMDS) ordination was used to observe community clusters associated with Bray-Curtis dissimilarity calculations. NMDS plots were used to survey bacterial OTU heterogeneity relative to that in each treatment, and permutational multivariate analysis of variance (PERMANOVA) comparisons of the Bray Curtis dissimilarity distances were performed. Next, the linear discriminant analysis (LDA) effect size (LEfSe) method, performed with the Galaxy application, was used to support high-dimensional class comparisons in order to determine the bacterial genus most likely to explain differences between groups (65). Finally, microbial association networks were evaluated using the Sparse Correlations for Compositional Data method (SparCC; SpiecEasi package) (22). Correlations among genera were shown in network graphs plotted with ggplot2 and formatted with ggpubr or with qgraph. Vegan (v. 2.5-6) was used for Shannon diversity, specnumber, adonis, and Bray-Curtis dissimilarity matrices. All *P* values from multiple comparisons were adjusted with false-discovery rate (FDR) method. All analyses were conducted in R version 4.0.3 (http://www.r-project.org). To characterize the five most abundant OTU sequences generated from the 16S rRNA gene amplicon data sets at the microbial species level, BLAST analysis was performed against the NCBI 16S rRNA gene database. In essence, sequences were identified at the species level if a fully sequenced or classified type species sequence matched the OTU with the highest bit score. When this was not possible, species-level assignments were based on full-length Sanger 16S rRNA gene sequencing of isolates, as described below.

### Isolation and identification of L. johnsonii and E. faecalis by 16S gene Sanger sequencing.

*Lactobacillus* species isolates (*n* = 5) were recovered on Rogosa agar after anaerobic incubation of tongue homogenates for 3 to 5 days (66). Presumed *Enterococcus* isolates (*n* = 10) were recovered on citrate-azide-Tween-carbonate (CATC) base agar, which has high selectivity for enterococci, after anaerobic incubation for 24 h (67). Individual colonies were restreaked to purity at least three times. Isolates were also selected on the basis of colony and cell morphology after Gram staining and stored in 20% glycerol at −80°C. Isolates were identified at the species level by Sanger sequencing of the entire 16S rRNA gene. Briefly, *Lactobacillus* and *Enterococcus* isolates were grown from glycerol stocks overnight in MRS or BHI broth at 37°C and 5% CO_2_. Total DNA was extracted from overnight cultures using the DNeasy blood and tissue kit (Qiagen) and used as the template in PCR. PCR was performed using GoTaq green (Promega, Madison, WI, USA) to amplify the entire 16S gene with the following primers: 16S_27F, 5′-AGA GTT TGA TCM TGG CTC AG-3′; 16S_1391R, 5′-GAC GGG CGG TGT GTR CA-3′. PCR products were sent to Eurofins (Louisville, KY, USA) for Sanger sequencing. Sequences were analyzed using SnapGene software (GSL Biotech LLC, San Diego, CA, USA). NCBI-BLASTN searches were performed in the 16S rRNA gene sequence database, optimized for highly similar sequences using a strict MegaBLAST search. The sequences of all *Lactobacillus* isolates were identified as Lactobacillus johnsonii (99.37% identity and maximum bit-score of 2,155 with strain CIP 103620; 99.10% identity and maximum bit-score of 2,131 for strain ATCC 33200, second best). All *Enterococcus* isolates were identified as Enterococcus faecalis (99.19% similarity and maximum bit-score of 2,139 for strains NBRC 1000481 and ATCC 19433). Sequence similarity among L. johnsonii isolates ranged between 99.2% and 99.6% and among E. faecalis isolates between 97.5% and 99.5%, suggesting that they may represent different strains.

### Effect of L. johnsonii on E. faecalis and C. albicans growth.

Murine L. johnsonii (MT-LB4) and E. faecalis (Ef-13) isolates were grown to early stationary phase anaerobically in MRS broth overnight, washed in phosphate-buffered saline (PBS), and adjusted to a starting cell density with an optical density at 600 nm (OD_600_) of 0.2 (∼5 × 10^7^ cells ml^−1^) in MRS broth. To assess the effect of L. johnsonii on E. faecalis growth, microorganisms were incubated alone or in combination (at 1:1 cell ratio) in 5 ml MRS broth supplemented with either 2% glucose or 2% sucrose, anaerobically for 24 h. Aliquots (100 μl) of these cultures were then plated in triplicates on Rogosa (for L. johnsonii) or CATC (for E. faecalis) agar plates and incubated anaerobically for up to 3 days for CFU counts.

To assess the effect of L. johnsonii on C. albicans growth, isolate MT-LB4 was grown in MRS broth overnight as described above. C. albicans SC5314 was grown aerobically in YPD broth overnight in an orbital shaker at 30°C, washed in PBS, and adjusted to 5 × 10^4^ cells ml^−1^ using a Neubauer chamber. The two microorganisms were inoculated alone or together in 5 ml MRS broth, with or without 2% dextrose, and incubated aerobically at 37°C in 5% CO_2_ for up to 24 h. Aliquots (100 μl) were collected every 90 min, plated in triplicates, and incubated anaerobically on MRS agar (for lactobacilli) or aerobically on Sabouraud dextrose agar supplemented with 10 μg ml^−1^ of chloramphenicol (for *Candida*) for CFU determinations. To assess if C. albicans inhibition was due to nutrient depletion by lactobacilli, fresh MRS media were added at each time point following aliquot removal for plating. In dose-response experiments, 10-fold serial dilutions of L. johnsonii cultures were tested with cell densities corresponding to 1:1 to 1:1,000 fungal/bacterial cell ratios.

To assess the effect of L. johnsonii on the ability of C. albicans to grow as biofilms, microorganisms were inoculated alone or together in 8-well slides, ibiTreat 15 μ-Slide wells (ibidi USA, Fitchburg, WI), in RPMI medium supplemented with 10% BHI and 10% fetal bovine serum (FBS), optimized for biofilm growth of *Candida* with other lactic acid bacterial species (68). L. johnsonii and C. albicans were seeded at 10:1 bacterial/fungal cell ratio and incubated overnight. To visualize *Candida*, calcofluor white was added 5 min prior to imaging with a fluorescence microscope. The medium pH was monitored at the beginning and end of the coculture period.

### Statistical analyses.

For most experimental outcomes, statistical significance was determined by two-tailed *t* test, assuming equal variances, or the Mann-Whitney test when data were not normally distributed, in GraphPad Prism 9 (GraphPad Software, Inc., La Jolla, CA). Hyphal length data were analyzed using nonparametric Kruskal-Wallis test followed by Dunn’s multiple-comparisons test. For microbiome sequence data, sample size estimation and power calculations were conducted using the R micropower package (69), using the distribution of pairwise Jaccard distances based on presence/absence of most abundant species from a set of preexisting samples. Based on PERMANOVA partitions, within-group and between-group distances allowed the calculation of the effect of each experimental group treatment upon the sampled microbiome. Simulations showed that our experiments were sufficiently powered to observe differences in pairwise distances and PERMANOVA comparisons among mouse groups (see Fig. S1 in the supplemental material).

### Data availability.

All 16s V4 DNA sequencing raw data have been deposited in NCBI, under SRA accession number PRJNA748424.

10.1128/mBio.01937-21.1 FIG S1 Microbiome PERMANOVA power estimation. This plot shows the power corresponding to different effect sizes simulated by segregating OTU membership, based on data from six groups with sample sizes ranging from 4 to 12 mice per group. Based on our data, the calculated ω^2^ was around 0.163, shown by the blue dotted line, and the corresponding power on the *y* axis was around 0.9. Based on our data set, this study was adequately powered across the existing groups for PERMANOVA comparisons. Download FIG S1, TIF file, 0.1 MB. Copyright © 2021 Bertolini et al. 2021 Bertolini et al. https://creativecommons.org/licenses/by/4.0/ This content is distributed under the terms of the Creative Commons Attribution 4.0 International license.
