# Supplementary figures and images for: Mucosal Bacteria Modulate Candida albicans Virulence in Oropharyngeal Candidiasis
Source: mBio. 2021 Aug 17;12(4):e01937-21. doi: 10.1128/mBio.01937-21 (PMC8406182; doi:10.1128/mBio.01937-21)

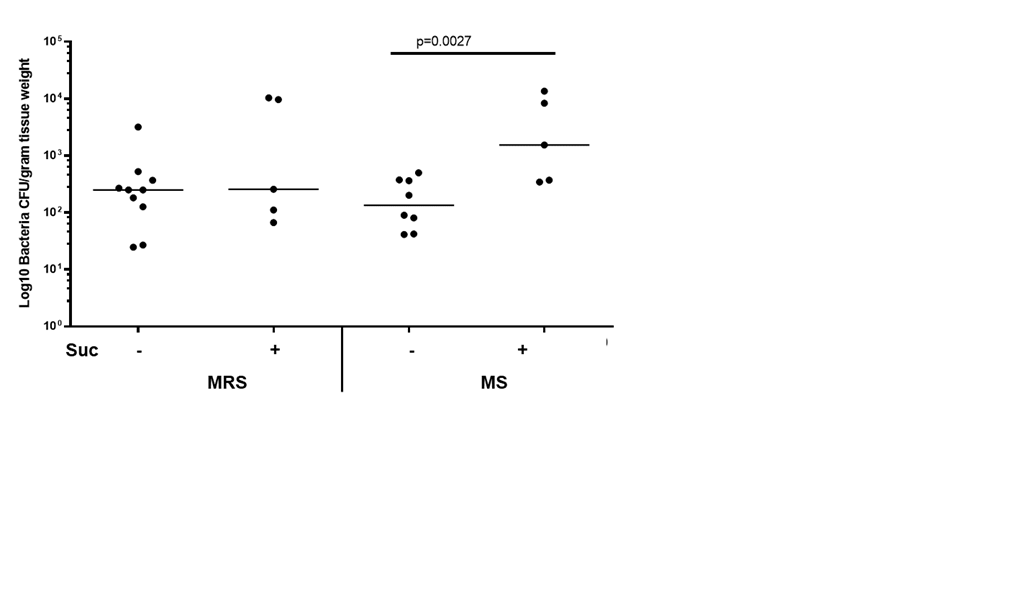

Supplement: FIG S2 [file mbio.01937-21-sf002.tif]

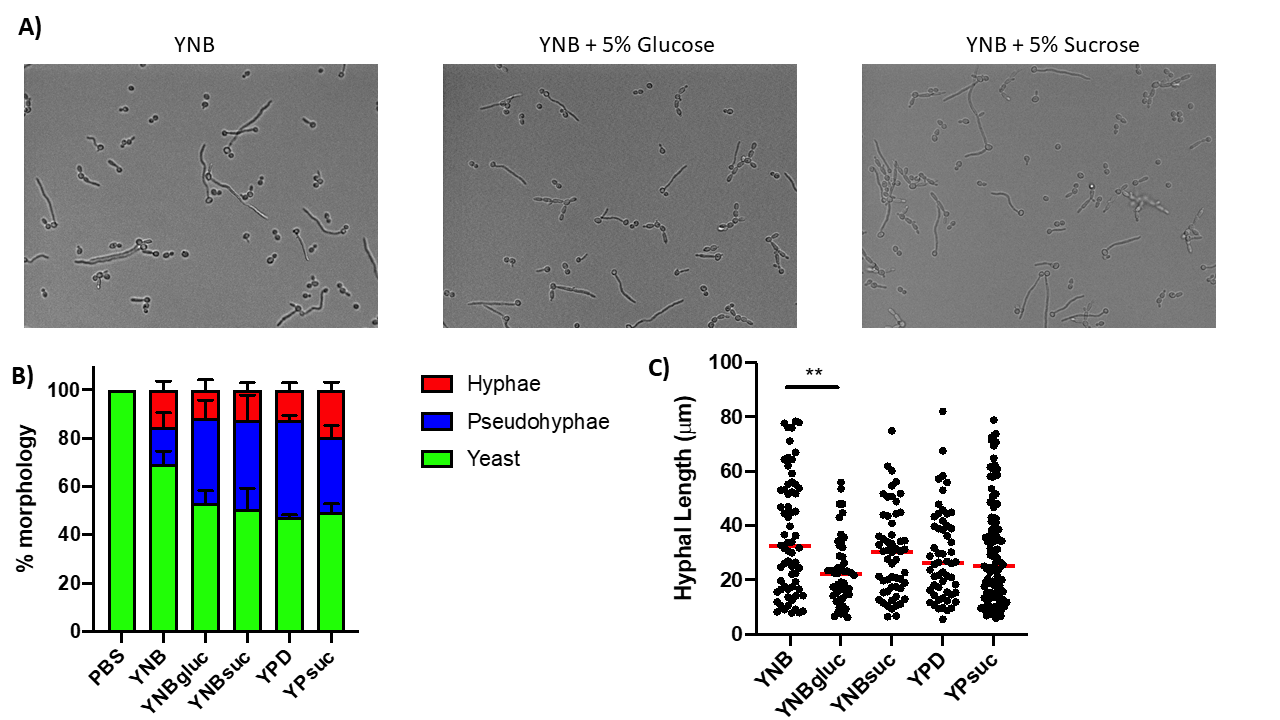

Supplement: FIG S3 [file mbio.01937-21-sf003.tif]

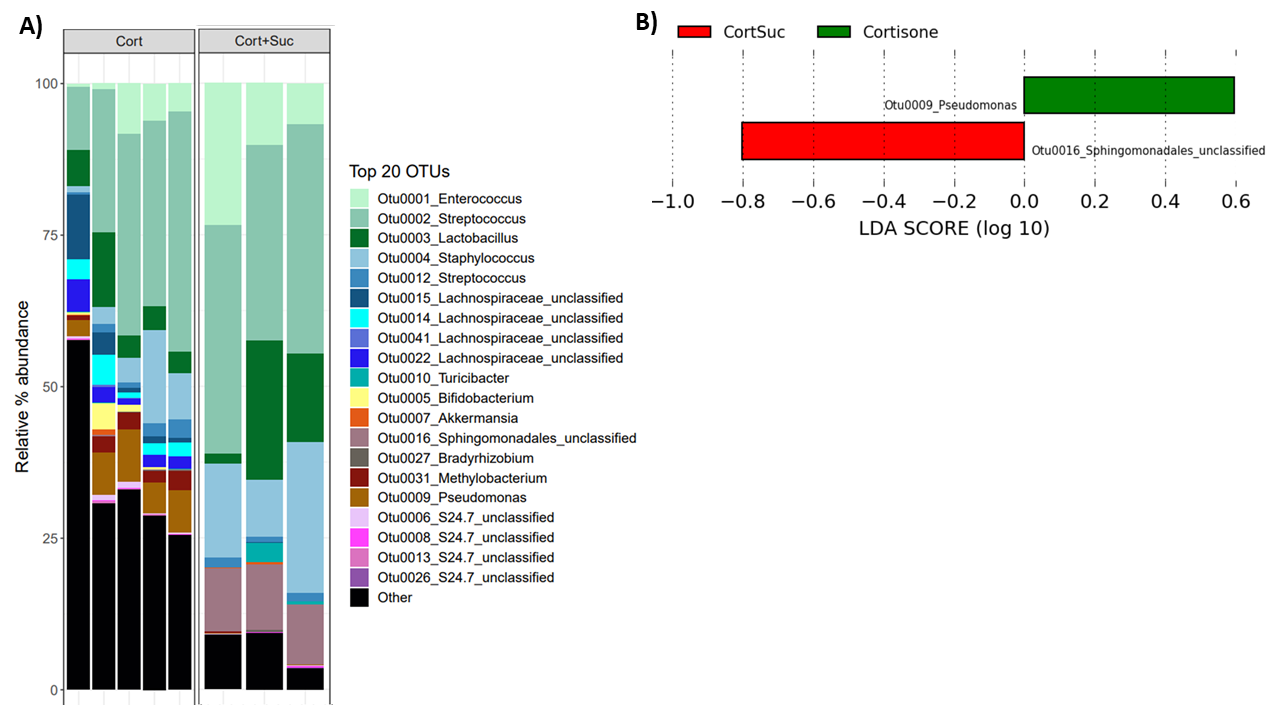

Supplement: FIG S4 [file mbio.01937-21-sf004.tif]

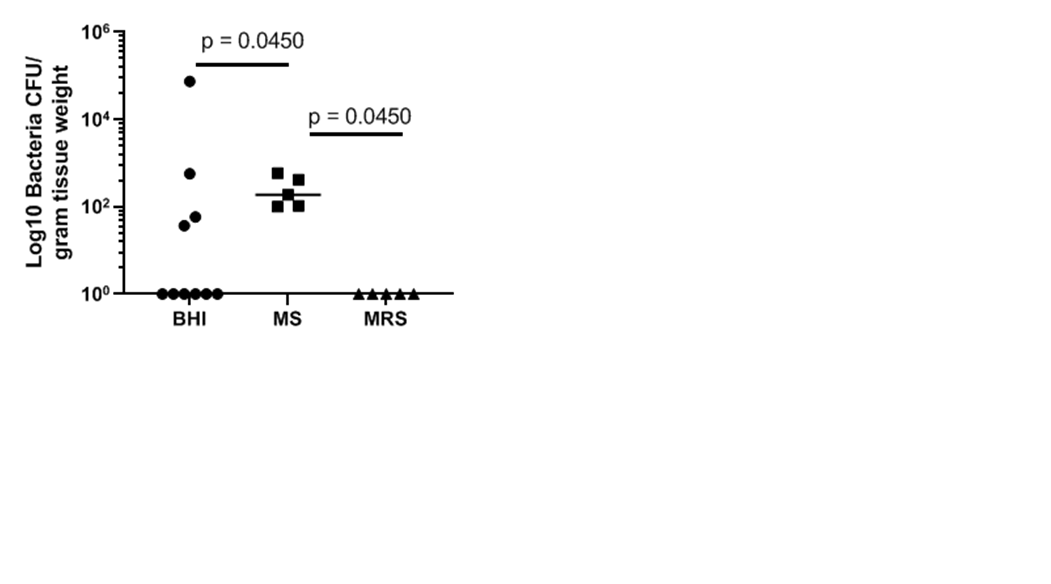

Supplement: FIG S5 [file mbio.01937-21-sf005.tif]

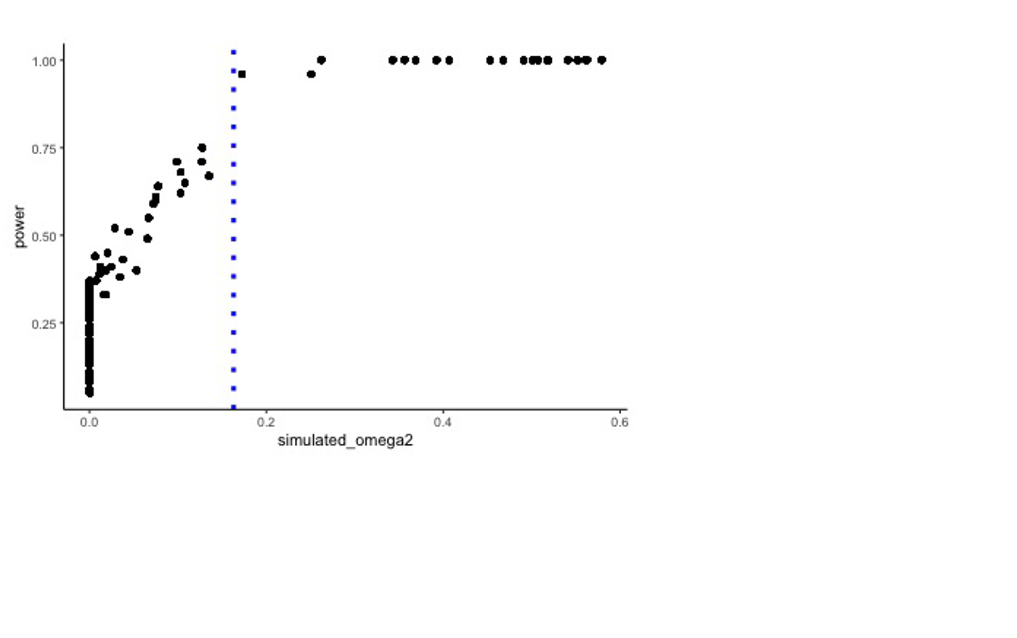

Supplement: FIG S1 [file mbio.01937-21-sf001.tif]
